# Supplementary material for: Network analysis of depression symptoms and physical activity levels before and after menopause
Source: PLoS One. 2025 Jun 23;20(6):e0325965. doi: 10.1371/journal.pone.0325965 (PMC12185023; doi:10.1371/journal.pone.0325965)
Supplement: S1 File — Contains 12 supplementary figures (S1–S12 Figs) and 4 supplementary tables (S1–S4 Tables) supporting the network analysis of depressive symptoms in pre- and postmenopausal women, including centrality stability, edge weight bootstrap tests, and model comparisons. (ZIP) [file pone.0325965.s001.zip › Supplementary documents.doc]

**Supplementary Information**

Supplementary Figure 1. The flow chart of the study

Supplementary Table 1. Mean, standard deviation, minimum, maximum, skewness, and kurtosis by the PHQ-9

Supplementary Figure 2. Stability of Network Centrality Index Before and After Menopause

Supplementary Figure 3. Bootstrap confidence intervals of edge weights in pre- and postmenopausal networks

Supplementary Figure 4. Differential Testing of Edge Strength in Networks Before and After Menopause

Supplementary Figure 5. The stability of the centrality index of depression symptoms network with different physical activities before menopause

Supplementary Figure 6. Bootstrap confidence intervals of edge weights for the network of depressive symptoms with different physical activity before menopause

Supplementary Figure 7. Differential Testing of Edge Strength in Depression Symptom Networks Across Different Physical Activities Before Menopause

Supplementary Figure 8. Standardized estimates of network centrality for depressive symptoms after menopause by physical activity level.

Supplementary Figure 9. Bootstrap confidence intervals for edge weights of networks of depressive symptoms with different physical activity after menopause

Supplementary Figure 10. Differential Testing of Edge Strength in Depression Symptom Networks with Different Physical Activities After Menopause

Supplementary Table 2. Analysis of correlation between Ising and GGM models

Supplementary Table 3. Global Intensity Comparison between Ising Model and GGM Model

Supplementary Figure 11. GGM Model Depressive Symptom Network

Supplementary Figure 12. Adjustment for covariates Depressive Symptoms Network

Supplementary Table 4. Analysis of correlation between original and adjusted covariate models

Supplementary Figure 1. The flow chart of the study

Supplementary Table 1. Mean, standard deviation, minimum, maximum, skewness, and kurtosis by the PHQ-9

| Depressive symptoms | PHQ-9 item | Min | Max | M | SD | Skewness | Kurtosis |
| --- | --- | --- | --- | --- | --- | --- | --- |
| Anhedonia | PHQ01 | 0 | 3 | 0.43 | 0.79 | 1.92 | 2.96 |
| Sad Mood | PHQ02 | 0 | 3 | 0.41 | 0.75 | 2.01 | 3.62 |
| Sleep | PHQ03 | 0 | 3 | 0.71 | 0.99 | 1.26 | 0.35 |
| Energy | PHQ04 | 0 | 3 | 0.90 | 0.96 | 0.91 | -0.11 |
| Appetite | PHQ05 | 0 | 3 | 0.51 | 0.86 | 1.69 | 1.89 |
| Guilt | PHQ06 | 0 | 3 | 0.31 | 0.70 | 2.53 | 5.92 |
| Concentration | PHQ07 | 0 | 3 | 0.30 | 0.71 | 2.57 | 6.03 |
| Motor | PHQ08 | 0 | 3 | 0.19 | 0.57 | 3.45 | 12.09 |
| Suicide | PHQ09 | 0 | 3 | 0.05 | 0.28 | 6.90 | 54.92 |

A

B

Supplementary Figure 2. Stability of Network Centrality Index Before and After Menopause

A

B

Supplementary Figure 3. Bootstrap confidence intervals of edge weights in pre- and

postmenopausal networks

Note: The red line represents the edge weight value, and the gray area is 95% CI.

A

B

Supplementary Figure 4. Differential Testing of Edge Strength in Networks Before and After Menopause

Note: Black boxes represent significant differences between the two nodes, while gray boxes indicate no significant differences between the two nodes.

A

B

Supplementary Figure 5. The stability of the centrality index of depression symptoms network with different physical activities before menopause

A

B

Supplementary Figure 6. Bootstrap confidence intervals of edge weights for the network of depressive symptoms with different physical activity before menopause

Note: The red line represents the edge weight value, and the gray area is 95% CI.

A

B

Supplementary Figure 7. Differential Testing of Edge Strength in Depression Symptom Networks Across Different Physical Activities Before Menopause

Note: Black boxes represent significant differences between the two nodes, while gray boxes indicate no significant differences between the two nodes.

A

B

Supplementary Figure 8. Standardized estimates of network centrality for depressive symptoms after menopause by physical activity level.

A

B

Supplementary Figure 9. Bootstrap confidence intervals for edge weights of networks of depressive symptoms with different physical activity after menopause

Note: The red line represents the edge weight value, and the gray area is 95% CI.

A

B

Supplementary Figure 10. Differential Testing of Edge Strength in Depression Symptom Networks with Different Physical Activities After Menopause

Note: Black boxes represent significant differences between the two nodes, while gray boxes indicate no significant differences between the two nodes.

Supplementary Table 2. Analysis of correlation between Ising and GGM models

| Item | | Edge | |
| --- | --- | --- | --- |
|  |  | r(95%CI) | P |
| Premenopausal | PA<600 | 0.938(0.417,0.665) | <0.001 |
|  | PA>=600 | 0.593(0.245,0.533) | <0.001 |
| Postmenopausal | PA<600 | 0.913(0.341,0.652) | <0.001 |
|  | PA>=600 | 0.764(0.330,0.783) | <0.001 |

Supplementary Table 3. Global Intensity Comparison between Ising Model and GGM Model

| Item | | Global Strength | | P |
| --- | --- | --- | --- | --- |
|  |  | Ising | GGM |  |
| Premenopausal | PA<600 | 23.499 | 4.009 | <0.001 |
|  | PA>=600 | 18.048 | 4.045 | <0.001 |
| Postmenopausal | PA<600 | 21.753 | 3.882 | <0.001 |
|  | PA>=600 | 23.793 | 3.765 | <0.001 |

Supplementary Figure 11. GGM Model Depressive Symptom Network

Note:Left A is the premenopausal depressive symptom network and right A is the postmenopausal depressive symptom network.

Left B is the premenopausal PA <600 depressive symptom network,right B is the premenopausal PA ≥600 depressive symptom network.

Left C is the postmenopausal PA <600 depressive symptom network, right C is the postmenopausal PA ≥600 depressive symptom network.

Supplementary Figure 12. Adjustment for covariates Depressive Symptoms Network

Note:Left A is the premenopausal depressive symptom network and right A is the postmenopausal depressive symptom network.

Left B is the premenopausal PA <600 depressive symptom network,right B is the premenopausal PA ≥600 depressive symptom network.

Left C is the postmenopausal PA <600 depressive symptom network, right C is the postmenopausal PA ≥600 depressive symptom network.

Supplementary table 4. Analysis of correlation between original and adjusted covariate models

| Item | Premenopausal | | | | Postmenopausal | | | |
| --- | --- | --- | --- | --- | --- | --- | --- | --- |
|  | PA<600 | | PA>=600 | | PA<600 | | PA>=600 | |
|  | r(95%CI) | p | r(95%CI) | p | r(95%CI) | p | r(95%CI) | p |
| Edge | 0.900(0.799,0.965) | <0.001 | 0.923(0.869,0.960) | <0.001 | 0.851(0.738,0.952) | <0.001 | 0.630(0.460,0.823) | <0.001 |
| Strength | 0.777(0.683,0.967) | 0.024 | 0.931(0.848,0.994) | <0.001 | 0.825(0.785,0.995) | 0.008 | 0.322(0.211,1.022) | 0.397 |
