# Supplementary material for: Network analysis of depression symptoms and physical activity levels before and after menopause
Source: PLoS One. 2025 Jun 23;20(6):e0325965. doi: 10.1371/journal.pone.0325965 (PMC12185023; doi:10.1371/journal.pone.0325965)
Supplement: Attachment — Submitted filename: Response to Reviewers.docx [file pone.0325965.s003.docx]

Dear Editor and reviewers :

Thank you very much for the professional advice given by the editor and review experts, we have made changes and additions to the issues raised by the editor and the reviewing experts.The specific modifications are as follows:

**Response to reviewer 1:**

**1.Some important papers are missing and need to be reviewed and discussed, such as，**

**Martin-Key, N. A., Funnell, E. L., Benacek, J., Spadaro, B., & Bahn, S. (2024). Using network analysis to understand the association between menopause and depressive symptoms. npj Women's Health, 2(1), 41.**

**Wen, J., Wang, W., Liu, K., Sun, X., Zhou, J., Hu, H., ... & Miao, M. (2024). The psychological side of menopause: evidence from the comorbidity network of menopausal, anxiety, and depressive symptoms. Menopause, 10-1097.**

**Cai, H., Chen, M. Y., Li, X. H., Zhang, L., Su, Z., Cheung, T., ... & Xiang, Y. T. (2024). A network model of depressive and anxiety symptoms: a statistical evaluation. Molecular psychiatry, 29(3), 767-781.**

**Fausto, D. Y., Leitao, A. E., Silveira, J., Martins, J. B., Dominski, F. H., & Guimaraes, A. C. (2023). An umbrella systematic review of the effect of physical exercise on mental health of women in menopause. Menopause, 30(2), 225-234.**

**Soares, C. N. (2019). Depression and menopause: an update on current knowledge and clinical management for this critical window. Medical Clinics, 103(4), 651-667.**

**For the paragraph following the introduction of network analysis in the introduction, it is important to review the current available evidence to explore the related topics using network analysis.**

We sincerely appreciate the reviewer's insightful suggestion regarding the integration of network analysis literature. As recommended, we have substantially revised the introduction section to incorporate recent advancements in network analysis applied to menopausal mental health.

**2.“These symptoms significantly impact society, affecting Manuscript approximately 350 million people globally.” This statement needs references.**

We would like to thank the reviewers for their suggestions.We have revised it in the text.

**3.Qgraph needs reference：**

**Epskamp S, Cramer AOJ, Waldorp LJ, Schmittmann VD, Borsboom D. qgraph: network Visualizations of Relationships in Psychometric Data. J Stat Softw. 2012;48(4):1–18. doi:10.18637/jss.v048.i04**

We sincerely appreciate the advice given by the reviewers and I have made changes in the article

**4.Since there are no length limits of plos one, thus, the important information in the supplementary material should be included in the formal context.**

We would like to thank the reviewers for their suggestions.We have revised it in the text.

**5.Please provide effect sizes where available.**

We would like to thank the reviewers for their suggestions.We have revised it in the text.

**6.The theoretical implications should be discussed in more details.**

We would like to thank the reviewers for their suggestions.We have revised it in the text.

**7.It should be noted that there are many demographic differences between Premenopausal and Postmenopausal groups and this is also the same case for PA<600 and PA≥600 groups were revealed. These should be included as Covariates in the analysis.**

We sincerely thank the reviewers for highlighting this critical methodological consideration. As suggested, we are adding this analysis to the final results section.

**Response to reviewer 2:**

**1.1.Please provide reliability coefficients (e.g., Cronbach's alpha) to evaluate the internal consistency and stability of your collected data.**

We sincerely thank the reviewers for their suggestions, which I have elaborated on in the Methods section PHQ9's Cronbach's alpha

**2.There are several formatting and typographical errors that need correction. For instance, why is "N" capitalized in "BootNet". Also, "BootNe" is missing the "t" in section 2.4.3. Similar formatting and spelling errors appear elsewhere in the manuscript.**

We would like to thank the reviewers for their suggestions. “BootNet” is a program package for the r-language, which I have modified in the text.

**3.In section 3.2, second paragraph, instead of using the qualitative statement "Sadness holds a central role," please specify which nodes exhibit the highest strength indices with their corresponding values. This would provide more precise and quantifiable results.**

We would like to thank the reviewers for their suggestions.We have revised it in the text.

**4.The use of the Ising model requires further justification. While you express concerns about modeling ordinal data, it should be noted that most network analyses of PHQ-9 utilize the GGM model, which has been confirmed as appropriate for this type of data by network analysis methodology developers.**

We would like to thank the reviewers for their suggestions.Given that the Gaussian graphical model (GGM) is premised on the continuous normal distribution of data, it is noteworthy that the PHQ9 data in this study are uniformly right-skewed (Supplementary Table 1). This skewness can result in inaccurate estimations of the covariance matrix when using the GGM model. Furthermore, the GGM model exhibits sensitivity to extreme values, which could potentially lead to either an underestimation or overestimation of correlations between variables. For the construction of the network model, this study opted for the "Ising" algorithm, wherein circular nodes symbolize depressive symptoms and the connecting lines between nodes, referred to as edges, have thicknesses indicative of the magnitude of the partial correlation coefficient.

**5.I recommend reporting only the Strength centrality index rather than all three indices (Strength, Closeness, and Betweenness). Consequently, the discussion section should focus on interpreting findings based on Strength centrality. This would enhance the clarity and interpretability of your results.**

We would like to thank the reviewers for their suggestions.We have revised it in the text.

6. **In your comparison of physical activity networks between pre- and post-menopausal women, the NCT results indicate no significant differences. Your statement that "The network model of PA<600 is more complex than that of PA≥600" requires precise**

We would like to thank the reviewers for their suggestions.We have revised it in the text.

**7.The manuscript requires comprehensive language editing, particularly regarding network analysis terminology. Please ensure consistent and precise usage of technical terms throughout the text.**

We would like to thank the reviewers for their suggestions.We have revised it in the text.

We have also made changes to other inappropriate parts of the original manuscript, including but not limited to: language presentation, number of references, and writing error issues. All of the above changes are highlighted in red in the article.

We are very grateful to the editors and reviewers for their careful guidance. Due to the limitation of academic level, if there are any inappropriate or imprecise areas, we hope you can give us your advice, and we will revise them carefully in time.

Sincerely yours,

Song Chen
