# Supplementary material for: Network analysis of depression symptoms and physical activity levels before and after menopause
Source: PLoS One. 2025 Jun 23;20(6):e0325965. doi: 10.1371/journal.pone.0325965 (PMC12185023; doi:10.1371/journal.pone.0325965)
Supplement: Attachment — Submitted filename: Response to Reviewers.doc [file pone.0325965.s004.doc]

Dear Editor and reviewers :

Thank you very much for the professional advice given by the editor and review experts, we have made changes and additions to the issues raised by the editor and the reviewing experts.The specific modifications are as follows:

**Response to reviewer #2:**

**1.Use of the Ising model: While the authors have explained their rationale for choosing the Ising model over the GGM model, this justification needs strengthening. The skewed distribution of data is typically not sufficient justification for dichotomizing data and employing the Ising model. I suggest that the authors:**

**Provide specific references supporting this methodological choice**

**Or, alternatively, consider conducting analyses using the GGM model as well, and comparing the results from both methods**

**This would enhance the robustness of the research methodology and provide readers with a more comprehensive analytical perspective.**

Thank you very much for the suggestions you made, and we fully recognize the importance of transparency and robustness in model selection for the credibility of the study's conclusions. In response to your comments, we have revised the manuscript.

**2.Inconsistent figure citation format: Throughout the manuscript, there is inconsistency in how figures are referenced. Some instances use "Figure 3" while others use "Fig. 1" or "Fig. 2". I recommend adopting a uniform citation format, preferably following PLOS ONE's formatting guidelines. This consistency would improve the professionalism and readability of the article.**

**Inconsistent p-value reporting: The reporting of p-values in the text lacks uniformity, with some values presented in the exact format "p=0.016" and others in the range format "P<0.05". I suggest the authors standardize the p-value reporting format and ensure consistency in capitalization (either consistently using lowercase "p" or uppercase "P").**

We would like to thank the reviewers for their suggestions.We have revised it in the text.

We have also made changes to other inappropriate parts of the original manuscript, including but not limited to: language presentation, number of references, and writing error issues. All of the above changes are highlighted in red in the article.

We are very grateful to the editors and reviewers for their careful guidance. Due to the limitation of academic level, if there are any inappropriate or imprecise areas, we hope you can give us your advice, and we will revise them carefully in time.

Sincerely yours,

Song Chen
