# Supplementary material for: Network analysis of depression symptoms and physical activity levels before and after menopause
Source: PLoS One. 2025 Jun 23;20(6):e0325965. doi: 10.1371/journal.pone.0325965 (PMC12185023; doi:10.1371/journal.pone.0325965)
Supplement: Attachment — Submitted filename: renamed_20868.doc [file pone.0325965.s005.doc]

Dear Editor :

Thank you very much for the professional advice given by the editor experts, we have made changes and additions to the issues raised by the editor experts.The specific modifications are as follows:

**Response to Editor Comments:**

**Line 141, page 3, please expand "MET" at first occurrence**

**Line 142, page 3, expand "PA" at first occurrence**

**Lines 144-145. page 3: Please consider rephrasing this sentence: "Physically active, while the remaining subjects were divided into low-intensity PA and high-intensity PA based on PA."**

**In Table 1: Please expand "RIDAGEYR" "RIDRETH1" and "DMDMARTL" as a foot note.**

**Line 426, page 14: Please expand "NCT" and abbreviate in parenthesis.**

We would like to thank the editor for their suggestions.We have revised it in the text.

We have also made changes to other inappropriate parts of the original manuscript, including but not limited to: language presentation, number of references, and writing error issues. All of the above changes are highlighted in red in the article.

We are very grateful to the editors and reviewers for their careful guidance. Due to the limitation of academic level, if there are any inappropriate or imprecise areas, we hope you can give us your advice, and we will revise them carefully in time.

Sincerely yours,

Song Chen
